# Cross-validation of chemical and genetic disruption approaches to inform host cellular effects on *Wolbachia* abundance in *Drosophila*

**DOI:** 10.3389/fmicb.2024.1364009

**Published:** 2024-03-25

**Authors:** Zinat Sharmin, Hani Samarah, Rafael Aldaya Bourricaudy, Laura Ochoa, Laura Renee Serbus

**Affiliations:** ^1^Department of Biological Sciences, Florida International University, Miami, FL, United States; ^2^Biomolecular Sciences Institute, Florida International University, Miami, FL, United States; ^3^Department of Chemistry and Biochemistry, Florida International University, Miami, FL, United States

**Keywords:** *Wolbachia*, *Drosophila*, titer, Wnt, mTOR, autophagy, endosymbiosis, commensalism

## Abstract

**Introduction:**

Endosymbiotic *Wolbachia* bacteria are widespread in nature, present in half of all insect species. The success of *Wolbachia* is supported by a commensal lifestyle. Unlike bacterial pathogens that overreplicate and harm host cells, *Wolbachia* infections have a relatively innocuous intracellular lifestyle. This raises important questions about how *Wolbachia* infection is regulated. Little is known about how *Wolbachia* abundance is controlled at an organismal scale.

**Methods:**

This study demonstrates methodology for rigorous identification of cellular processes that affect whole-body *Wolbachia* abundance, as indicated by absolute counts of the *Wolbachia surface protein* (*wsp*) gene.

**Results:**

Candidate pathways, associated with well-described infection scenarios, were identified. *Wolbachia*-infected fruit flies were exposed to small molecule inhibitors known for targeting those same pathways. Sequential tests in *D. melanogaster* and *D. simulans* yielded a subset of chemical inhibitors that significantly affected whole-body *Wolbachia* abundance, including the Wnt pathway disruptor, IWR-1 and the mTOR pathway inhibitor, Rapamycin. The implicated pathways were genetically retested for effects in *D. melanogaster*, using inducible RNAi expression driven by constitutive as well as chemically-induced somatic GAL4 expression. Genetic disruptions of *armadillo*, *tor,* and *ATG6* significantly affected whole-body *Wolbachia* abundance.

**Discussion:**

As such, the data corroborate reagent targeting and pathway relevance to whole-body *Wolbachia* infection. The results also implicate Wnt and mTOR regulation of autophagy as important for regulation of *Wolbachia* titer.

## Introduction

Resident intracellular microbes, referred to as endosymbionts, are widespread in nature. Endosymbiotic microbes are commonly thought of as mutualists, in which the interaction between host and microbe benefits both partners of the symbiosis. However, endosymbionts can also exhibit relatively inert (commensal) or detrimental (parasitic) interactions with a host organism. Evidence suggests that some commensal and/or mutualistic microbes are descendants of formerly parasitic ancestors (Sachs et al., 2014). Other endosymbionts have been found to exhibit context-dependent plasticity in their symbiotic interactions, as seen in *Salmonella*, which is carried innocuously by chickens, but causes severe infection in humans (Foley et al., 2013). To account for this diversity, intracellular bacteria are now described in terms of a symbiotic spectrum, ranging from mutualistic to parasitic (Lewis, 1985).

For any endosymbiont, high infection prevalence in host populations is the mark of success. Members of the *Wolbachia* genus are naturally widespread bacterial endosymbionts, carried in certain lineages of mites, crustaceans, nematodes and in about 50% of all insect species (Sazama et al., 2019). *Wolbachia* are often described as reproductive parasites because some strains induce parthenogenesis, male-killing, feminization or cytoplasmic incompatibility, which ultimately favor the success of infected females (Werren et al., 2008). In other instances, *Wolbachia* have been found to serve as mutualists, by sustaining host viability and reproduction (Taylor et al., 2005; Pannebakker et al., 2007; Landmann et al., 2011), as well as by repelling harmful viral infections in the host (Hedges et al., 2008; Teixeira et al., 2008; Cogni et al., 2021). Due to absence of evident benefits or detriments, *Wolbachia* infections can often be described as commensal. The *Wolbachia-*host symbiosis thus provides a new and valuable perspective for investigating, defining, and understanding the cellular basis of commensalism.

To date, *Wolbachia* studies have shared an interest in understanding how endosymbiont amount (titer) is specified within tissue culture cells, dissected host tissues, and whole organisms. *Wolbachia* titer has been assessed as a function of various host/strain combinations as well as in response to host age, crowding, temperature, diet, genetic background, microbiota, and chemical exposure (Hoffmann et al., 1998; Veneti et al., 2004; Unckless et al., 2009; Wiwatanaratanabutr and Kittayapong, 2009; Voronin et al., 2012; Ali et al., 2019; López-Madrigal and Duarte, 2019). A patchwork of cytological and qPCR-based methods have been used across assessments of *Wolbachia* abundance *in vivo*, with shared recognition that cellular processes interacting with *Wolbachia* may also affect *Wolbachia* abundance within the host (López-Madrigal and Duarte, 2019). The field is now in a position to investigate more broadly how *Wolbachia-*host interactions inform mechanisms of infection.

Fundamental questions remain regarding the involvement of host cellular processes in endosymbiotic infection. It is not clear whether signaling pathways relevant to *Wolbachia* infection have been fully identified, nor which relays of those relays affect *in vivo Wolbachia* titer most strongly. It also remains unclear what mechanistic attributes distinguish commensal infections from detrimental scenarios. To this end, this study asked whether *Wolbachia* titer is affected by the same host cellular pathways as commonly studied bacterial infections. 14 candidate host pathways and processes were tested, using complementary chemical and genetic tools. Whole-body *Wolbachia* abundance was assessed by real-time qPCR, to determine absolute counts of the *Wolbachia surface protein* (*wsp*) gene. This work yielded a subset of host functions for further pursuit, with implications for the basis of commensalism.

## Materials and methods

### *Drosophila* stocks and maintenance

Two fly strains were used in this study. Preliminary screening was performed using *Drosophila melanogaster* of the genotype *w*; *Sp/Cyo*; *Sb/TM6B* carrying the endogenous *w*Mel *Wolbachia* strain (Serbus and Sullivan, 2007; Christensen et al., 2016). *Drosophila simulans (D. sim)* infected with the endogenous *Wolbachia riverside* (*w*Ri) strain were used for further analyses (Hoffmann et al., 1990; Serbus and Sullivan, 2007). GAL4 lines sourced from the Bloomington Drosophila Stock Center (BDSC) were also used to drive RNAi expression. Constitutive somatic expression was driven by the Actin5C-GAL4 driver *w; P{Act5C-GAL4-w}E1/Cyo* (BDSC# 25374) and the daughterless-GAL4 driver *w; P{w+, GMR12B08-GAL4}attP2* (BDSC# 48489). Mifepristone-induced gene expression was driven by the GeneSwitch-GAL4 driver *yw {hs-FLP}; {w+, UAS-GFP}; {w+, Act5C-GS-GAL4}/TM6B, Tb* (BDSC #9431).

Fruit flies were maintained in plastic bottles/vials containing standard fly food media. The recipe was derived from Bloomington Drosophila Stock Center as described previously (Christensen et al., 2016). The flies were raised in an Invictus *Drosophila* incubator at 25°C, under standard 12:12 h light–dark cycle. For the experiments, “0-day old” flies were collected and kept on standard fly food medium for 2 days. The flies were then used for drug treatments in vials or within a plate assay format as described previously (Christensen et al., 2019). Only female flies were used for the plate-based screening experiments, to reduce possible variation in population behavior per well.

### Chemical food preparation

Two or more chemicals were used to alter the functionality of each of the candidate host processes pursued in this study. Where possible, compounds with opposite effects on the process of interest were included, such as the microtubule-depolymerizing drug, colchicine, and the microtubule-stabilizing drug, taxol, as well as the phospholipase C (PLC) inhibitor, U73122, and the PLC activator, 3-M3MFBS. The final list included a total of 37 candidate compounds (Supplementary Table S1). All the drugs were dissolved in DMSO. Most stock solutions, including rifampicin, were prepared in advance as 10 mM solutions, aliquoted, and stored at −20°C. Rapamycin was ordered as a 5 mM solution in DMSO, also stored at −20°C. Light-sensitive drugs were stored in the dark.

Immediately before use, chemical stocks were thawed and diluted 100X into fly food that had been re-melted, then cooled. Control vials, prepared in parallel with the chemical treatment vials, were treated with equivalent amounts of DMSO alone. In all cases, the final concentration of DMSO in food was capped at 1%. For the chemical screen, a minimum of 10 mL drug food was prepared per condition, to be further dispensed in approximately 1 mL amounts per treatment well. For drug lethality tests and *GS-GAL4* induction experiments that were carried out in vials, food containing control DMSO and DMSO-solubilized compounds was prepared in larger volumes, to be dispensed into vials as 5 mL final amounts. After pouring, plates and vials were cooled and solidified in the fume hood, with foil wrappings used to protect light-sensitive compounds. Treatment vials were stored in Ziplock bags at 4°C as needed.

For the chemical screen,10 female flies were transferred to each treatment well. A DMSO-solubilized rifampicin control was also run on every qPCR plate to confirm the ongoing capacity of *Wolbachia* to respond to compound treatments. After 3 days of chemical feeding, pools of 5 female flies were removed from each treatment well and processed as a group for *wsp* quantification using real-time qPCR. For the vial-based experiments that assessed drug lethality, flies were incubated in groups of 12, 6 females and 6 males per vial, with viability scored every 3 days. Flies were transferred to new treatment vials at day 6, using vials from the 4°C fridge that had been re-warmed. For vial-based experiments using DMSO and mifepristone, flies were incubated on treatment food in groups of 15 females and 5 males. Flies were transferred to new treatment vials every 3 days, using DMSO and mifepristone vials from the 4°C fridge that had been re-warmed. After 14 days of feeding were completed (Haselton et al., 2010; Serbus et al., 2015), pools of 5 female flies were removed from each vial and processed as a group for *wsp* quantification using real-time qPCR.

### Genetic manipulations

To incorporate *Wolbachia* into GAL4 driver lines, the driver males were crossed to virgin females of the genotype *w*; *Sp/Cyo*; *Sb/TM6B,* carrying the *w*Mel *Wolbachia* strain (Christensen et al., 2016), which in this study is referred to as DB *w*Mel. F1 progeny were backcrossed to the parental lines to establish *Wolbachia-*infected driver stocks, with the same genotypes as the originally uninfected lines.

Genetic disruptions were achieved using VALIUM20 transgenic RNAi lines which depend on short hairpin RNA, also known as artificial microRNAs, to trigger gene silencing in both somatic and germline cells (Ni et al., 2011). Each host pathway was tested by two different UAS-shRNA responder lines (Supplementary Table S2), selected in accord with pathways targeted by “hit” compounds from the chemical screen. To generate RNAi-expressing flies, *w*Mel-infected virgin females were selected from freshly emerging bottles of each GAL4 driver stock. These females were crossed to males that carried responder UAS (upstream activating sequence) elements adjacent to a promoter that drives shRNA production (Ni et al., 2011). The parent flies were removed from the vials after 3–4 days of mating. Emerging F1 flies were collected in daily cohorts and aged for 5 days. The F1s that carried the GAL4 driver and the UAS responder were identified by phenotypic markers and separated within each cohort. Control siblings that contained either the GAL4 or UAS responder, but not both, were collected when available. In some cases, a separate control set was also generated in parallel by outcrossing *Wolbachia-*infected driver females to Oregon R (OreR) males. In all cases, control and treatment groups were generated and processed in parallel for *wsp* quantification.

### DNA extraction and qPCR for whole-body *Wolbachia* quantification

Real-time qPCR was used to assess whole-body *Wolbachia* abundance, using the candidate gene *wsp* as a proxy for *Wolbachia* genomes per sample. Because *Wolbachia* reportedly carry one genome per bacterial cell (McGarry et al., 2004), resulting genome counts are expected to represent *Wolbachia* abundance per sample. DNA was extracted from pools of 5 female flies as per established methods (Christensen et al., 2019). Absolute measurements of the *wsp* gene from the extracted DNA samples were compared against reference plasmid standards, specifically a PGEM-T vector carrying a 160 bp PCR-amplified fragment of the *wsp* gene (Christensen et al., 2016). Real-time qPCR was carried out on a Bio-Rad CFX96 Connect Optics Module Real-Time System. Absolute *wsp* copy numbers were obtained by comparing cycle threshold (Ct) values to the standard curve generated from the plasmid standard. The *wsp* amplification primers were: Fwd 5’ CATTGGTGTTGGTGTTGGTG 3′ and Rev. 5’ ACCGAAATAACGAGCTCCAG 3′, used at 5 μM (Christensen et al., 2016).

### Data display and analyses

Graphical displays showing “normalized” *wsp* counts as a scatter plot were created for display purposes only. To generate such graphs, median *wsp* counts for the DMSO controls per replicate were identified, then compared to the median *wsp* count of the entire dataset. A scaling factor was then identified and applied to each replicate, to normalize the median *wsp* value for the DMSO control and all associated experimental data. The raw absolute count data are available for review as needed (Supplementary material S1). Statistical analyses were conducted on raw (non-normalized) data within each experimental replicate for all experiments. Statistics appropriate to data normality and homogeneity were identified and applied as previously (Christensen et al., 2019). Power analysis was performed with an alpha set at 0.05 using a MATLAB-based data sub-sampling program, designed by Dr. Philip K. Stoddard. This program has the advantage that analyses can be customized to the statistical test appropriate to each dataset (Christensen et al., 2019). All statistical analysis worksheets for each experiment performed are also available (Supplementary material S1).

## Results

### Identifying and targeting candidate host processes relevant to bacterial infection

A literature search was first conducted to assess how intracellular bacterial abundance is regulated in commonly studied bacterial infections. After assessing 52 species from 17 genera, 26 bacterial species were identified, for which host gene/pathway effects on density regulation had been discussed (Supplementary Table S3). Of these, the literature highlighted 14 host mechanisms that altered the intracellular abundance of multiple bacterial classes (Supplementary Table S4; Supplementary material S2). Because these mechanisms were identified as more commonly involved in host–microbe interactions, they were prioritized for testing in the *Wolbachia-Drosophila* endosymbiosis model. Candidate compounds known to target each process were selected, with two or more compounds identified for testing each of the 14 classes of host targets. This culminated in the selection of 37 total candidate compounds to test for effects on whole-body *Wolbachia* titer (Table 1).

**Table 1:** Screening targets and chemical tools.

Candidate host processes	Drug name	Drug effect
Cell cycle modulation	Flavopiridol	CDK inhibitor
	Roscovitine	CDK inhibitor
	Sodium butyrate	HDAC inhibitor
Cytoskeleton-based transport	Colchicine	Microtubule destabilizer
	Cytochalasin D	F-actin destabilizer
	Taxol	Microtubule stabilizer
Ubiquitin-proteasome system	Bortezomib	Proteasome inhibitor
	Epoxomicin	Proteasome inhibitor
Mitochondrial/ Antioxidant	Butylated hydroxytoluene	Antioxidant
	L-Glutathione	Antioxidant
	MitoBloCK-6	Mitochondrial import inhibitor
	Niclosamide	Pink1 activator
	Resveratrol	Cox-1 inhibitor
Apoptotic pathway	ABT-199	Bcl-2 inhibitor
	Apoptosis inhibitor	Caspase 3 inhibitor
	Caspase 8 inhibitor	Caspase 8 inhibitor
Imd pathway	CAY10512	NF κ B inhibitor
	SC76741	NF κ B inhibitor
GPCR signaling	Caffeine	cAMP phosphodiesterase inhibitor
	SQ22536	Adenylyl cyclase inhibitor
Phospholipase-related	M-3M3FBS	Phospholipase C activator
	U73122	Phospholipase C inhibitor
Calcium signaling	Nicardipine HCl	L-type voltage-dependent calcium channel inhibitor
	Verapamil	L-type calcium channel blocker
Ras/mTOR pathway	Erlotinib HCl	EGFR inhibitor
	Trametinib	MEK inhibitor
	Rapamycin	mTOR inhibitor
	Wortmanin	PI3 kinase inhibitor
Nitric oxide synthase	L-NAME	Nitric oxide synthetase inhibitor
	Methylxanthine	cAMP and cGMP phosphodiesterase inhibitor
	Sildenafil citrate	cGMP phosphodiesterase inhibitor
Jak–Stat signaling	Ruxolitinib	JAK 1/2 inhibitor
	SH-4-54	STAT2 inhibitor
Wnt pathway	IWR-1	AXIN inhibitor
	Ly090314	GSK-3 inhibitor
Kinase modulator	Pyrvinium pamoate	Akt inhibitor
	Staurosporine	PKC inhibitor

### Host-directed small molecules alter *wsp* abundance in adult *Drosophila* hosts

**Figure 1:**
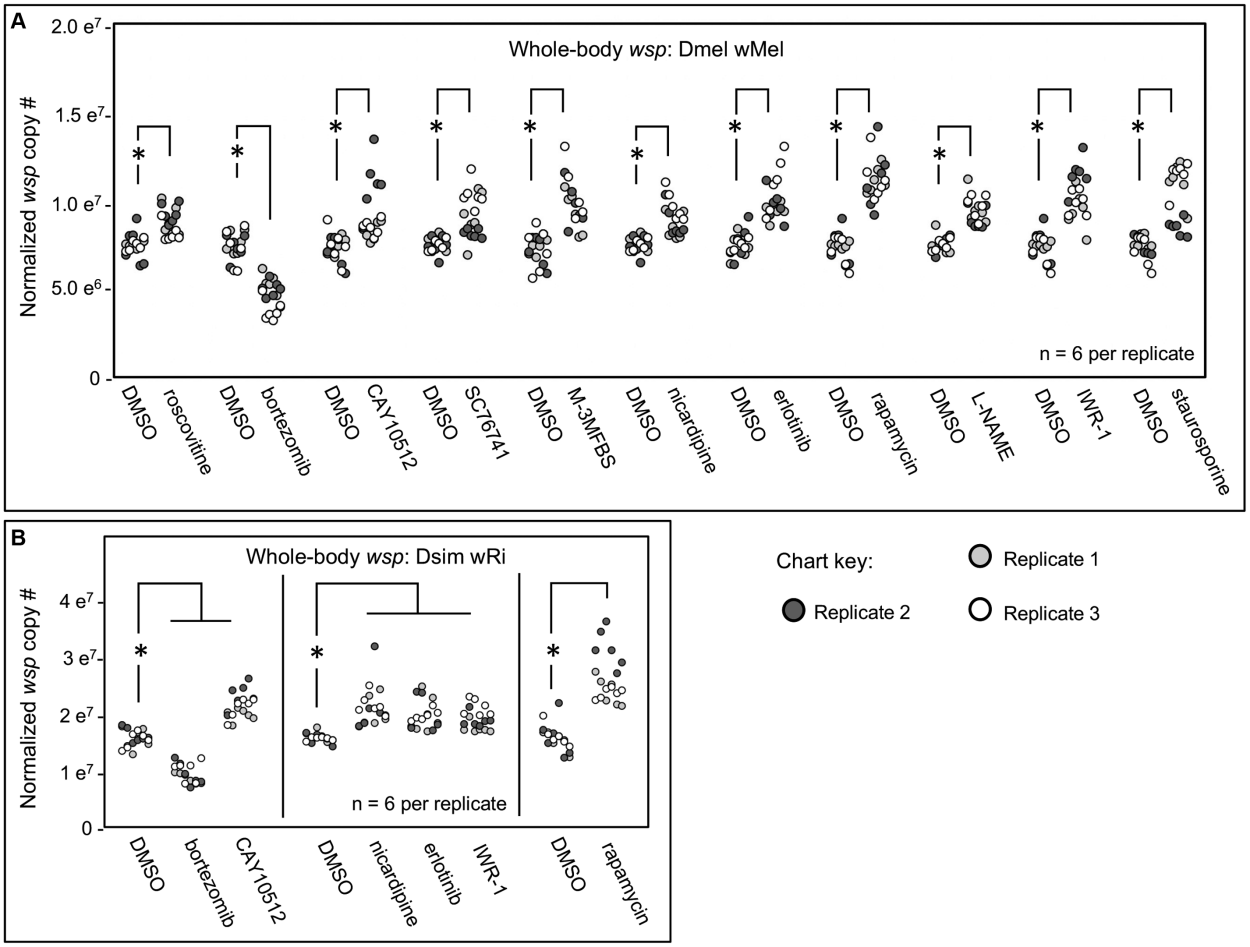
Whole body *wsp* abundance in response to chemical treatments. Display shows DMSO controls normalized across replicates, and the corresponding drug treatment data scaled accordingly. **(A)** Chemical treatment effects on whole-body *wsp* abundance in Dmel *w*Mel. **(B)** Chemical treatment effects on whole-body *wsp* abundance in Dsim *w*Ri. Significance was set at * *p* < 0.05, and is displayed only for conditions where all replicates met this standard.

The impact of the candidate compounds on whole-body *Wolbachia* titer was screened by absolute quantification of *wsp* by real-time qPCR (Kim et al., 2008; Serbus et al., 2012; Markstein et al., 2014; Christensen et al., 2019). *D. melanogaster* flies, carrying the endogenous *w*Mel *Wolbachia* strain, termed DB *w*Mel, were exposed for 3 days to food supplemented with DMSO-solubilized drugs or DMSO alone as a control. Treatments were initially tested for impact on whole-body *wsp* across two independent plate replicates. Those treatments which significantly changed *wsp* abundance in both plates were identified as preliminary hits. Of 37 chemicals tested, the primary screen identified 16 compounds were identified as meeting this criterion. These preliminary hit compounds were re-tested for reproducibility in a third plate replicate. 11 compounds were reconfirmed as hits, implicating a total of 9 host pathways and processes (Figure 1A). Most “hit” compounds elicited an increase in whole-body *wsp* abundance, with median values ranging 6–57% higher than the control (*p* < 0.001–0.036, *n* = 6 per plate replicate). The only exception was bortezomib, which reduced *wsp* to 48–71% of the DMSO control (*p* < 0.001, *n* = 6 amplifications per plate replicate) (Figure 1A; Table 2).

**Table 2:** Chemical screen outcomes: comparing Dmel *w* Mel hits to Dsim *w* Ri results.

Host cellular process	Drug name	*p*-value range for Dmel *w*Mel	*p*-value range for Dsim *w*Ri	Hit in both systems?
Cell cycle modulation	Roscovitine	0.003–0.009	0.006–0.283	No
Ubiquitin-proteasome system	Bortezomib	<0.001 (all)	<0.001 (all)	Yes
Imd pathway	CAY10512	0.001–0.036	<0.001–0.001	Yes
	SC76741	<0.001–0.022	0.171–0.936	No
Phospholipase-related	M-3M3FBS	0.002–0.004	0.009–0.840	No
Calcium signaling	Nicardipine HCl	<0.001–0.016	<0.001–0.006	Yes
Ras/mTOR pathway	Erlotinib HCl	<0.001–0.002	<0.001–0.024	Yes
	Rapamycin	<0.001–0.001	<0.001 (all)	Yes
Nitric oxide synthase	L-NAME	<0.001–0.002	0.001–0.056	No
Wnt pathway	IWR-1	<0.001–0.001	<0.001–0.041	Yes
Kinase modulator	Staurosprine	0.002–0.006	0.003–0.061	No

To investigate a role for candidate processes across systems, the hits from DB *w*Mel were retested against the *D. simulans* (Dsim) model, which naturally carries the *w*Ri *Wolbachia* strain. The Dsim *w*Ri re-screen identified a subset of 6 compounds that significantly affected whole-body *wsp* counts across 3 plate replicates (Figure 1B). 5 compounds increased whole-body *wsp* abundance to 15–52% higher than the DMSO control (*p*-value range: <0.001–0.041, *n* = 6 per plate replicate). These hits were associated with host Imd signaling, Calcium signaling, Ras/mTOR signaling, and Wnt signaling functions. By contrast, the proteasome inhibitor bortezomib continued to reduce *wsp* counts to 56–69% of the DMSO control (*p* < 0.001, *n* = 6 amplifications per plate replicate) (Figure 1B; Table 2).

**Figure 2:**
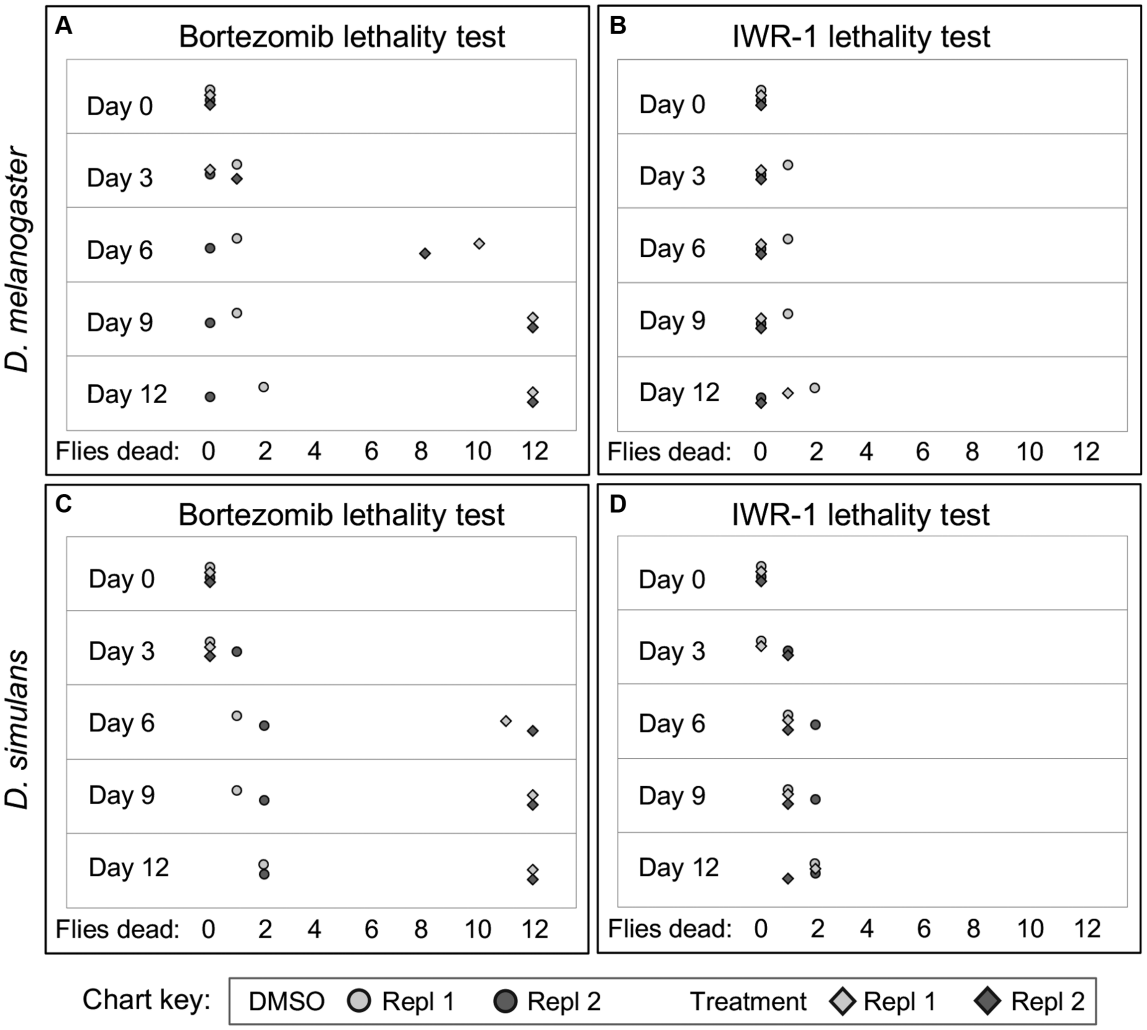
Example figure showing lethality data for bortezomib and IWR-1 compounds in *D. melanogaster* and *D. simulans*. **(A–D)** Depict the lethality effects two representative compounds, bortezomib **(A,C)** and IWR-1 **(B,D)**, on *D. melanogaster* and *D. simulans*. Each panel shows the number of dead flies at different time points (days) following exposure across two independent replicates. Circles: Control groups treated with 1% DMSO vehicle only. Diamonds: Experimental groups treated with bortezomib **(A,C)** or IWR-1 **(B,D)**.

To further investigate why some chemical treatments increase *wsp* counts, but others suppress *wsp,* a lethality assay was conducted. Flies were exposed to each of the “hit” compounds for a 12-day period. Bortezomib induced high lethality by the 6-day exposure timepoint for both DB *w*Mel and Dsim *w*Ri (Figures 2A,C). Thus, it is possible that *wsp* reductions by bortezomib reflect a *Wolbachia* response to toxic host conditions. However, flies exposed to all other “hit” compounds exhibited viability profiles comparable to DMSO controls, as per the example of IWR-1 (Figures 2B,D) (Supplementary Figure S1). Because the non-lethal “hit” compounds were all shown to elevate *wsp* counts, these results suggest that functions of multiple host pathways normally reduce whole-body *Wolbachia* loads.

### Constitutive RNAi disruptions corroborate a subset of host pathway effects on *wsp*

To confirm the basis for host cellular effects on whole-body *Wolbachia* titer, genetic disruption experiments were performed, focusing on the pathways dually implicated by chemical screening of DB *w*Mel and Dsim *w*Ri. We used the GAL4::UAS system in *D. melanogaster*, which enables directed manipulation of gene expression (Brand and Perrimon, 1993; Duffy, 2002). In this case, the GAL4::UAS system was set to drive expression of short hairpin RNAi suppress the corresponding gene product (Perrimon et al., 2010; Ni et al., 2011; Perkins et al., 2015). The *w*Mel strain was crossed into well-established GAL4 driver lines that drive constitutive whole-body expression, including the reputedly “strong” Actin5C-GAL4 driver (*Act-5C*), and the “milder” daughterless-GAL4 driver (*da-GAL4*) (Supplementary Table S5). Few to no F1 progeny were recovered that carried *Act5C-GAL4* as well as *UAS-shRNA* chromosomes, indicating lethality for such genetic combinations. However, crossing the *UAS-shRNA* lines to *da-GAL4* (Serbus et al., 2015) yielded ample RNAi-expressing F1 progeny for analysis.

No changes in *wsp* abundance were detected in response to constitutive shRNA disruption of Calcium signaling by knockdown of L-type calcium channels encoded by *Ca-alpha1D* and *Cac*. Inconsistent effects on *wsp* abundance were associated with disruptions to the Imd pathway by knockdown of NF-kappa-B/*Rel*, and the Rel activator, *Tak1*. Similar inconsistencies were observed for knockdown of the Wnt pathway gene, *shaggy (sgg)* gene (Supplementary material S1).

**Figure 3:**
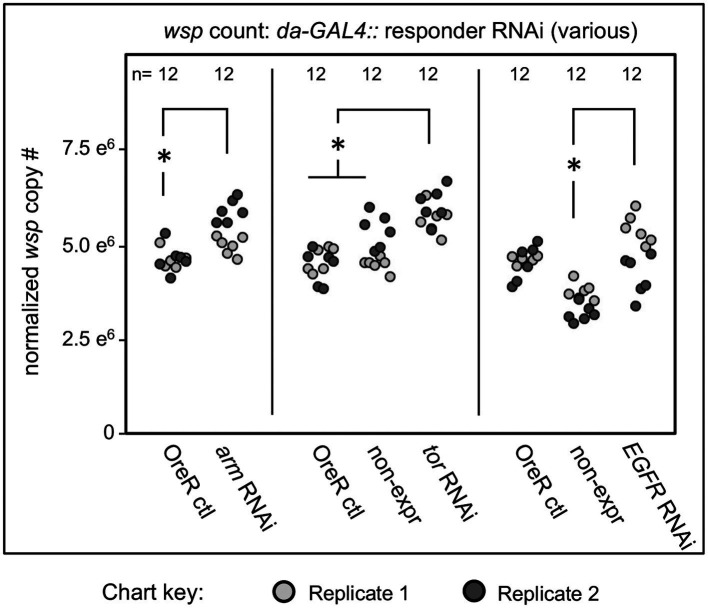
Whole body *wsp* abundance in control vs. *da-GALA*:*UAS-RNAi* knockdown flies. Oregon-R outcross controls are included throughout, and non-expressing sibling controls are shown where available. Display shows OreR controls normalized across replicates, and the corresponding conditions scaled accordingly. Panel shows data from 2 independent biological replicates. RNAi-expressing conditions shown from left to right are: *armadillo*, *tor*, and *EGFR* Significance was set at * *p* < 0.05, and is displayed only for conditions where all replicates met this standard.

Constitutive shRNA disruption of the Wnt pathway gene *armadillo (arm)* yielded positive effects, increasing median whole-body *wsp* counts to 9–15% above the OreR-outcrossed control (*p*-value range: <0.001–0.034, *n* = 6 per plate replicate) (Figure 3). A significant *wsp* increase was also detected for the Ras/mTOR pathway, with *tor* disruption flies exhibiting higher whole-body *wsp* counts at 23–31% above both sibling controls and OreR-outcrossed controls (*p* < 0.001, *n* = 6 per plate replicate) (Figure 3). Ras/mTOR signaling was also retested by knockdown of the *Epidermal Growth Factor Receptor (EGFR)*. Compared to OreR-outcrossed controls, *EGFR* knockdowns did not consistently affect *wsp* abundance measurements. However, in comparison to sibling controls, EGFR disruption yielded a 35–44% increase in median *wsp* counts (*p*-value range: <0.001–0.002, *n* = 6 per plate replicate) (Figure 3). Thus, detection of *wsp* responses to EGFR disruption may be context-dependent.

**Figure 4:**
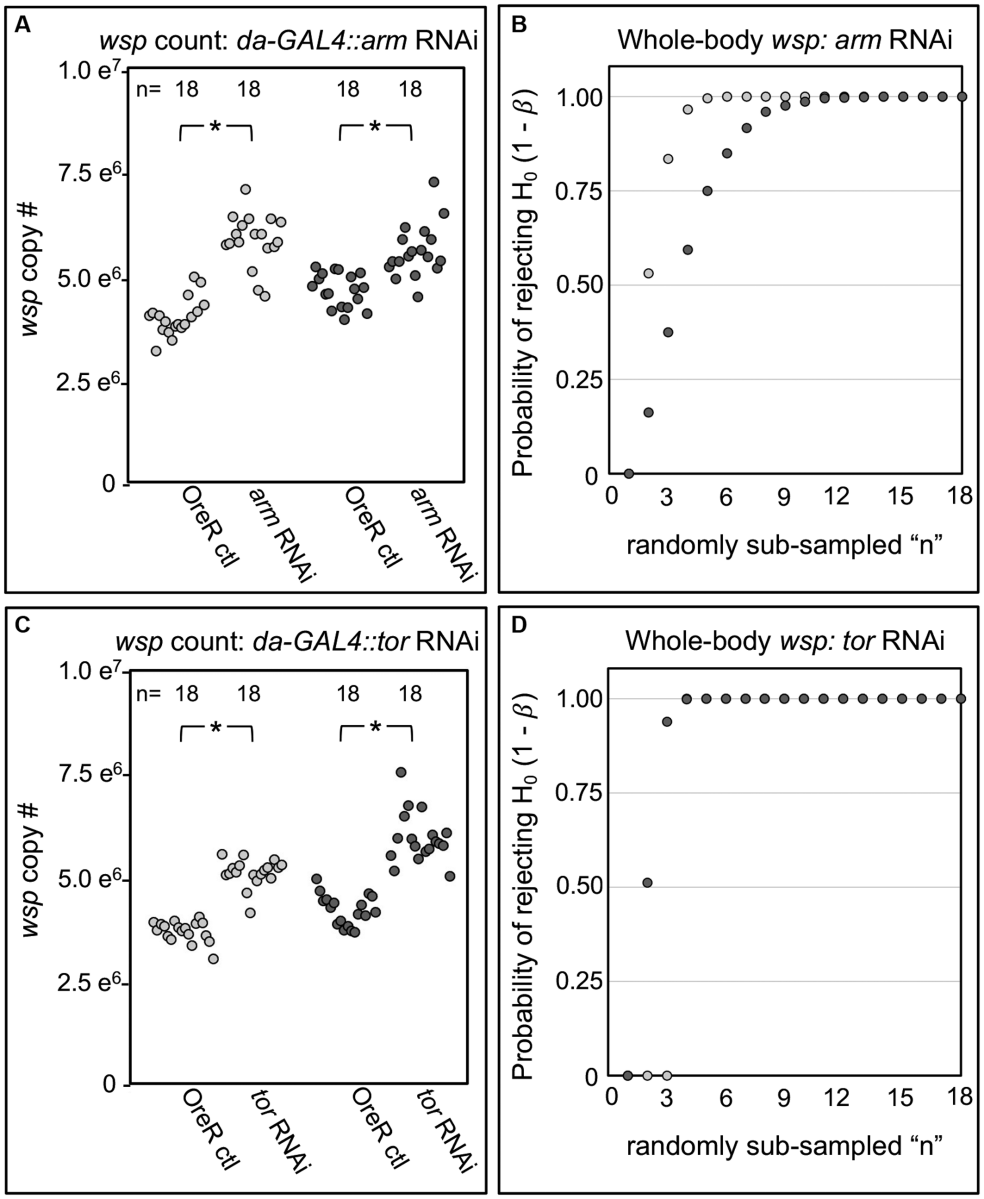
Whole body *wsp* abundance in control vs. *da-GAL4*::*UAS-RNAi* knockdown fies. Panels show data from 2 independent biological replicates. **(A)** Whole-body *wsp* abundance in *arm*-RNAi conditions. **(B)** Power analysis, testing the likelihood of significance as a function of sample size in **(A)**. **(C)** Whole-body *wsp* abundance in *tor*-RNAi conditions. **(D)** Power analysis, testing the likelihood of significance in **(C)**. Significance was set at * *p* < 0.05.

To confirm an effect of Wnt and Ras/mTOR pathways on *Wolbachia,* the most consistent outcomes from the *da-GAL4::UAS-shRNA* experiments were retested. *Arm* RNAi elicited a 16–50% increase in median *wsp* abundance over the OreR-outcrossed control (*p* < 0.001, *n* = 18) (Figure 4A). Power analysis indicated the *arm* RNAi outcome to be robust (β < 0.003 at *n* ≥ 12; total *n* = 18) (Figure 4B). *tor* RNAi triggered a 38–39% increase in *wsp* abundance as compared to OreR-outcrossed controls (*p* < 0.001, *n* = 18) (Figure 4C), a finding also well-supported by power analysis (β < 0.002 at *n* ≥ 4; total *n* = 18) (Figure 4D). Taken together, these data indicate that constitutive RNAi disruption of Wnt and Ras/mTOR signaling increases whole-body *Wolbachia* titer.

### Adult-induced RNAi disruptions provide further context for host effects on *wsp*

An intrinsic limitation of certain genetic disruption approaches, like constitutive RNAi induction, is that cumulative disruption effects could occur. To confirm ongoing Wnt and mTOR pathway effects on whole-body *wsp* abundance, “Gene-switch” GAL4 driver flies were used to induce GAL4 activity in adult flies. The Gene-switch version of GAL4 carries an inhibitory domain that blocks GAL4 function, until a de-repressor compound, mifepristone, is added (Roman et al., 2001). Because trial experiments on mifepristone identified low-power but potentially significant effects on *wsp* (Supplementary Figure S2), all *GS-GAL4* experiments were carried out with multiple controls. Flies carrying *GS-GAL4::UAS-shRNA* genotypes were always compared to non-expressing siblings, with half of the flies DMSO-treated, and the other half exposed to DMSO-solubilized mifepristone.

**Figure 5:**
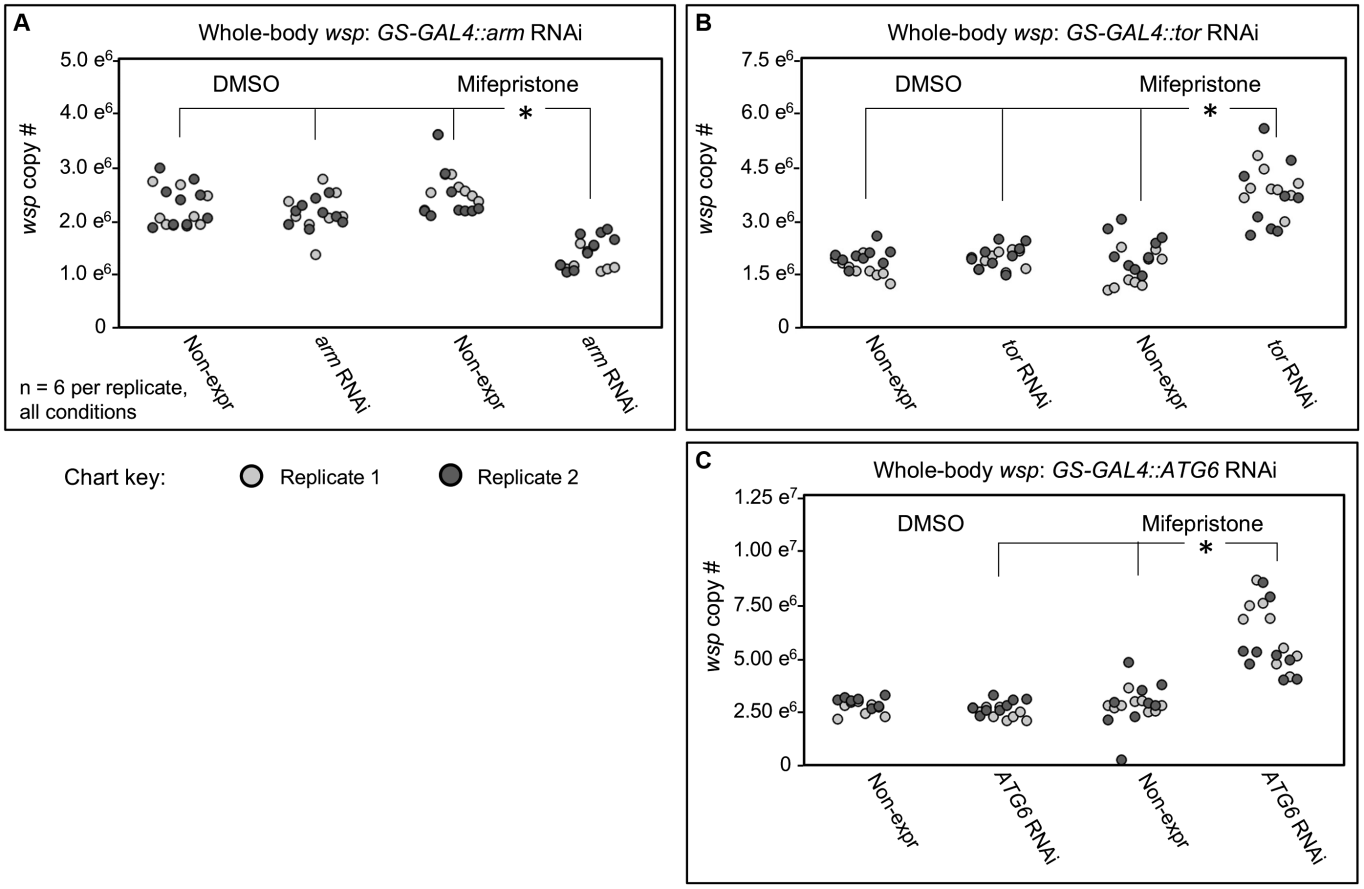
Whole body *wsp* abundance in control vs. *GS-GAL4*::*UAS-RNAi* knockdown flies. Flies capable of dsRNA expression were compared against non- expressing siblings, in the presence of DMSO or Mifepristone dissolved into DMSO. Genetic disruptions tested: **(A)** *arm* RNAi, **(B)** *tor* RNAi, **(C)** *ATG6* RNAi. Data out of range for **(C)**: 3 outliers for the Non-expressing DMSO condition at 2.01×10^7^, 2.54×10^7^, 2.064×10^7^, and 1 outlier for the ATG6 RNAi DMSO condition at 2.28×10^7^. Significance was set at * *p* < 0.05, and is displayed only for conditions where all replicates met this standard.

In tests of *arm* and *tor* RNAi knockdowns, no significant *wsp* abundance differences were observed between the DMSO-treated flies and mifepristone-treated flies that were incapable of RNAi expression (Figures 5A,B). However, mifepristone-fed flies that were capable of shRNA expression did show significant differences in their *wsp* counts. In the case of *GS-GAL4::arm-shRNA,* the mifepristone-treated flies exhibited reduced *wsp* counts, down to 45–71% of all other conditions tested in parallel (*p*-value range: <0.001–0.033, *n* = 9) (Figure 5A). By contrast, mifepristone-treated *GS-GAL4::tor-shRNA* flies carried 81–184% more *wsp* than all other conditions run in parallel (*p*-value range: <0.001–0.031, *n* = 9) (Figure 5B). These results confirm ongoing *Wolbachia* sensitivity to Wnt and mTOR disruption in adult hosts. Unlike the da-GAL4 data, the GS-GAL4 results notably show that Wnt and mTOR exert opposing effects on *Wolbachia* titer. This highlights a functional difference between constitutive and adult-specific disruptions of the Wnt pathway with respect to regulation of *Wolbachia* titer in adult insects.

One way to reconcile effects of *arm* and *tor* disruptions on *wsp* abundance is to consider the possibility that both may affect a consensus target relevant to *Wolbachia*. Literature indicates that Wnt signaling can suppress autophagy onset via multiple routes (Pérez-Plasencia et al., 2020), including through down-regulation of *Beclin-1*, also known as *ATG6* (Tao et al., 2017). mTORC1 is also known to inhibit ATG6 by suppressing the ATG6 activator, ULK1 (Hill et al., 2019). To test the effect of *ATG6* on whole-body *Wolbachia* titer, the *GS-GAL4::ATG6-shRNA* flies were generated. The mifepristone-fed, RNAi-expressing condition exhibit 67–173% higher *wsp* levels than non-expressing mifepristone-fed siblings and DMSO-fed flies of equivalent genotype (*p*-value range: <0.001–0.045, *n* = 9) (Figure 5C). These data suggest that *ATG6* normally suppresses whole-body *wsp* abundance, consistent with autophagy as a general suppressor of *Wolbachia* titer (Voronin et al., 2012; Strunov et al., 2022). Implications for Wnt and mTORC1 pathway interaction with autophagy are discussed below.

## Discussion

This study explored the basis for endosymbiosis by inipvestigating the effect of candidate host processes on *Wolbachia* titer in two different host-strain combinations. To identify consensus cellular effects on whole-body *wsp* counts, candidate compounds were screened against DB *w*Mel and Dsim *w*Ri systems. This was followed by constitutive as well as inducible genetic disruptions in DB *w*Mel to further verify effects of the drug-implicated pathways on *wsp* abundance. The amenability of *Drosophila* to mechanistic cross-validation in this rigorous capacity has opened a series of questions and opportunities, while also informing on the mechanisms of endosymbiont titer control.

After identifying infection-related pathways of potential interest from the literature, a candidate drug screen was performed to test these pathways. *Wolbachia* titer responses were assessed via absolute quantification of the *wsp* gene from whole insect samples. This is a targeted approach, relative to prior comprehensive screens of *Wolbachia-*host interactions in *Drosophila* tissue culture cells (White et al., 2017; Grobler et al., 2018). The organism-centered approach provides a unique advantage in detecting system-level, endogenous responses, with measurements inclusive of bacterial relocation events within the organism (Landmann et al., 2012; White et al., 2017). Detection of an organismal titer change is also a stringent requirement because *Wolbachia* infection is carried in a variety of tissues (Heddi et al., 1999; Bian et al., 2013; Ali et al., 2018; Schneider et al., 2018; Kaur et al., 2020), and it cannot be assumed that host manipulation will elicit uniform titer change across all tissues. Treatments yielding mild or contradictory outcomes at the tissue level will not be detected as hits by this method. Starting with a chemical screen provides an additional advantage in helping to narrow down the range of pathways for follow-up genetic testing, which as shown here, requires calibration at the level of tool selection, sample size, experiment duration, and controls required. Absolute counts by real-time qPCR are indispensible for the success of such analyses, to mitigate artifacts attributable to variable host ploidy, which may not always be foreseeable across tissues, systems, organism age, and nutritional conditions (Christensen et al., 2019; Ren et al., 2020).

This study emphasized pursuit of host pathways that are associated with commonly studied bacterial infections, as a springboard to delve deeper into processes which may also be involved in commensal *Wolbachia* infection. The cellular microbiology literature yielded a range of interesting host-side effects on bacterial genera such as *Coxiella, Legionella, Brucella, Rickettsia*, *Ehrlichia, Chlamydia*, and *Ehrlichia* (Rikihisa et al., 1995; Kessler et al., 2012; Czyż et al., 2014; Luo et al., 2016), for which host Calcium and Wnt signaling promotes bacterial proliferation. Another example is the Epidermal Growth Factor Receptor (EGFR) signaling pathway, which has been implicated in promoting host cell invasion by pathogens like *Salmonella* and *Neisseria* (Galán et al., 1992; Slanina et al., 2014). Other processes, such as the mTOR/autophagy pathway, have been shown to exert differential density effects depending on the bacterial strain. For example, mTOR signaling disruptions reduce intracellular loads for *Ehrlichia* (Luo et al., 2017), *Chlamydia, Listeria* (Derré et al., 2007), and *Salmonella* (Birmingham et al., 2006), but increase titers for *Anaplasma* and *Rickettsia* (Niu et al., 2008; Bechelli et al., 2018). Recurrent titer-related effects for host cellular processes on unrelated bacterial taxa invoke the possibility of generalized infection roles for host cellular pathways, and thus of potential interest in endosymbiosis as well (Supplementary Table S4) (Porter and Sullivan, 2023).

The candidate chemical screen yielded 11 compounds that consistently altered whole-body *wsp* levels in DB *w*Mel, 6 of which repeated the effect in Dsim *w*Ri. The “hit” compounds that were identified reflect roles for the Imd pathway, Calcium signaling, the Ras/mTOR pathway, and the Wnt pathway (Table 2), prioritizing these pathways for genetic follow-up experiments. The basis for a reduction in compound “hits” between DB *w*Mel to Dsim *w*Ri is inconclusive at this time. Some of the compounds may also have differential bioavailability, bioactivity and perdurance across systems, among other possibilities.

A notable aspect of the consensus, non-lethal hit compounds in both systems is that they all significantly increased *wsp* counts. Elevated *Wolbachia* titer has previously been observed in response to ribosome disruption, for example (Grobler et al., 2018). Perhaps *Wolbachia* suppression by a suite of host cellular processes is relieved by disruption to host functional networks, allowing a favorable shift in *Wolbachia* life cycle dynamics. It is reasonable to consider commensalism as an artifact of endosymbiont genome reduction, with virulence factors eliminated over time (Metcalf et al., 2014; Sachs et al., 2014; Latorre and Manzano-Marín, 2017). The findings of this study suggest that for *Wolbachia,* commensalism is further supported by ongoing containment of endosymbiont population by the host, consistent with a view that commensalism is not necessarily free of conflict (Keeling and McCutcheon, 2017).

**Figure 6:**
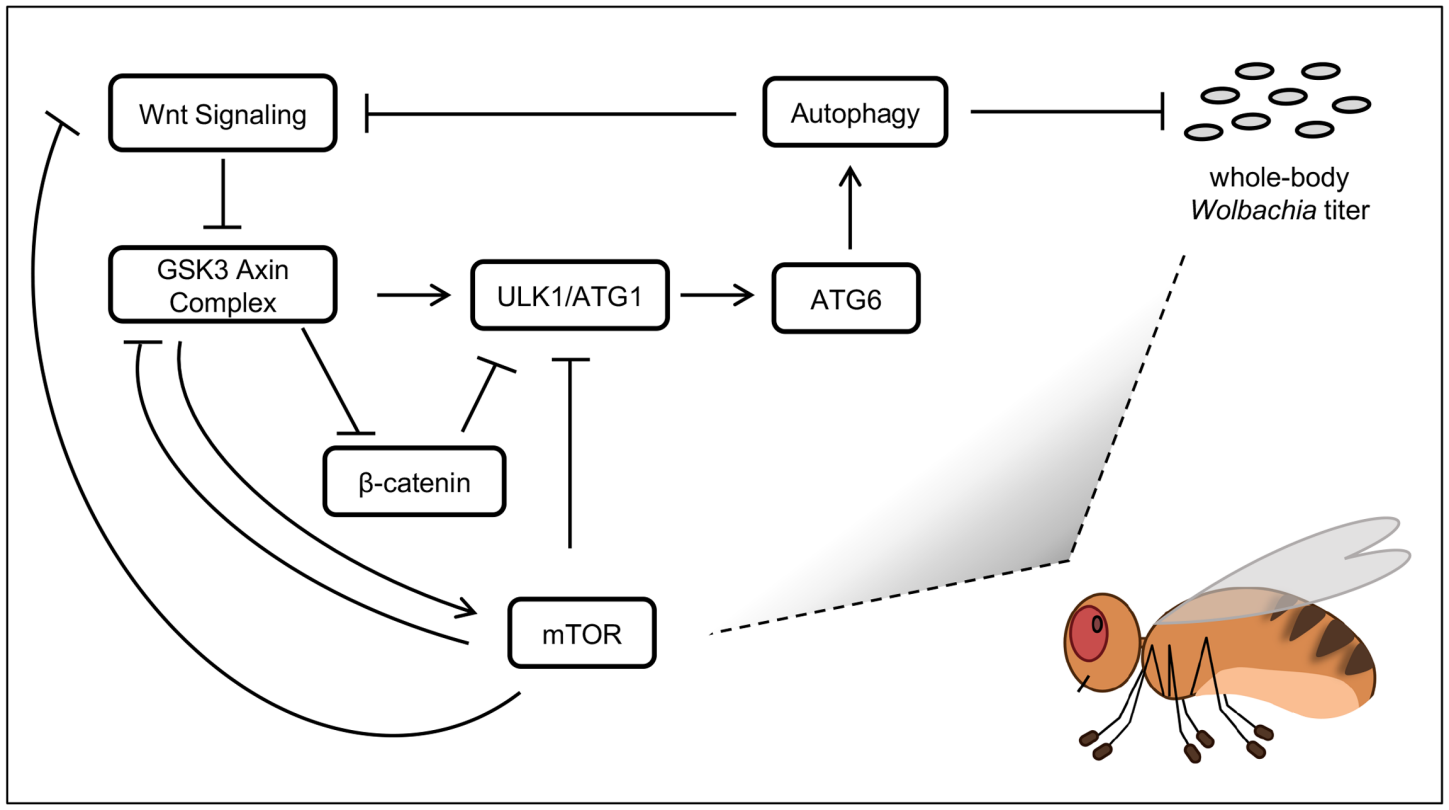
Models of host effects on whole-body *Wolbachia* titer. Pathway functions are displayed as per field literature. Boxes: host functions. Grey ovals: *Wolbachia* bacteria.

This study used complementary genetic approaches to cross-validate pathways implicated as *Wolbachia*-related by the chemical screen. Genetic corroboration of the host pathway functions is not always possible, as internal redundancies may render certain knockdowns ineffective. Developmental tolerance limits may also preclude analysis of the strongest knockdown effects, as when Actin5C-GAL4 was used in this study. Regardless of this, RNAi disruptions to host *arm* and *tor* genes affected whole-body *wsp* levels consistently and significantly. Dual effects of Wnt and mTOR disruptions on *Wolbachia* titer are consistent with literature connecting these signaling inputs to regulation of autophagy (Figure 6). There is a robust literature on antimicrobial functions of autophagy (Moy and Cherry, 2013), with reports of insects succumbing to pathogen infection when genes like ATG6 have been disrupted (Edosa et al., 2020).

Autophagy has been discussed previously as a regulator of endosymbiont titer, with functional effects dependent on the system used (Supplementary Table S6). Of the factors analyzed in this study, the one most immediate to the process of autophagy is ATG6 (Su et al., 2020). Thus, ATG6 suppression by RNAi would be expected to down-regulate autophagy. RNAi knockdowns of ATG6 yielded *Wolbachia* titer elevation, suggesting a model in which autophagy normally suppresses *Wolbachia* titer (Figure 6). This outcome is consistent with past results from others reporting that somatic autophagy antagonizes somatic *Wolbachia* titer (Voronin et al., 2012; Strunov et al., 2022).

The literature has also shown that the Wnt pathway affects autophagy regulation. In the absence of Wnt ligand, GSK-3 promotes autophagy activity by activating ULK1 (Ryu et al., 2021) as well as by suppressing Arm (Aros et al., 2021), which is a negative regulator of autophagy (Petherick et al., 2013; Feng et al., 2023) (Figure 6). Notably, GSK-3 works in a complex together with AXIN to suppress Arm (Ikeda et al., 1998), therefore AXIN disruption by IWR-1 should disrupt that function, with an indirect consequence of down-regulating autophagy, and allowing *Wolbachia* titer to increase. Tests of IWR-1 in this study yielded consistent *Wolbachia* titer elevation in both *D. melanogaster* and *D. simulans.* This finding is in accord with autophagy as a suppressor of whole-body *Wolbachia* abundance.

There is at least some complexity in Wnt pathway effects on whole-body *Wolbachia* titer. Because the GSK-3/AXIN complex antagonizes Arm in the Wnt pathway (Aros et al., 2021), Arm disruption would be expected to show the opposite results from an AXIN disruption. Meaning, since IWR-1 caused a titer increase, genetic disruptions in *arm* would be expected to prompt a titer decrease. From a mechanistic standpoint, this scenario would also make sense. It has been shown that Arm can suppress autophagy (Petherick et al., 2013; Feng et al., 2023) (Figure 6), so autophagy functions should increase when Arm RNAi is expressed, resulting in *Wolbachia* titer reduction. In this study, *Wolbachia* titer decreased in the induced *GS-GAL4:: Arm-shRNA* conditions. However, the constitutive *da-GAL4:: Arm-shRNA* treatment yielded a titer increase, not decrease. This disparity may be due to constitutive da-GAL4 disruption eliciting a cumulative, lifelong effect, with the possibility of involvement by other compensatory pathways. By contrast, the induction of GS-GAL4 in adults is uncoupled from earlier developmental events and may provide a more focused view of host pathway effects on *Wolbachia* titer in adult insects.

The mTOR pathway is well-known for regulation of autophagy. The mTORC1 complex has been shown to down-regulate autophagy through suppression of ULK1 (Yamamoto et al., 2023), as well as through suppression of GSK3 (Papadopoli et al., 2021) which would otherwise promote ULK1 function (Ryu et al., 2021) (Figure 6). In an alternate scenario, mTORC1 has the opposite regulatory effect, by inhibiting Wnt signaling at the level of the receptor, shutting down its function (Zeng et al., 2018). In that case, GSK3 would remain free to perform the complementary functions of activating ULK1 (Ryu et al., 2021) while also preventing Arm from suppressing ULK1 function (Petherick et al., 2013; Feng et al., 2023) (Figure 6). With this range of possibility, mTOR effects on autophagy function could go either way.

All mTORC1 disruptions in this study yielded a whole-body *Wolbachia* titer increase, regardless of the experimental system or type of manipulation tool used. This robust set of results is also compatible with the possibility of autophagy suppression of *Wolbachia*. If true, the implication would be that under normal conditions, mTORC1 function emphasizes suppression of Wnt receptor activity relative to other autophagy-related targets, to promoting autophagy and indirectly, *Wolbachia* titer suppression. This model comes with a grain of salt, as signaling processes can be complex. The reported finding that autophagy can down-regulate Wnt signaling (Pérez-Plasencia et al., 2020) is just one example of the nuance that may be involved (Figure 6). Future studies will be needed to elucidate how signaling and autophagy initiation affect *Wolbachia* and other microbial endosymbionts.

## Data availability statement

The original contributions presented in the study are included in the article/Supplementary material, further inquiries can be directed to the corresponding author.

## Ethics statement

The animal study was approved by FIU Institutional Biosafety Committee (IBC). The study was conducted in accordance with the local legislation and institutional requirements.

## Author contributions

ZS: Conceptualization, Data curation, Methodology, Supervision, Validation, Visualization, Writing – original draft, Writing – review & editing, Formal analysis, Investigation. HS: Conceptualization, Data curation, Formal analysis, Investigation, Methodology, Supervision, Validation, Visualization, Writing – review & editing. RB: Conceptualization, Data curation, Formal analysis, Investigation, Methodology, Validation, Visualization, Writing – review & editing. LO: Conceptualization, Data curation, Formal analysis, Investigation, Methodology, Validation, Visualization, Writing – review & editing. LS: Conceptualization, Data curation, Methodology, Validation, Visualization, Writing – review & editing, Funding acquisition, Project administration, Resources, Software, Supervision, Writing – original draft.
