# Supplementary material for: Cross-validation of chemical and genetic disruption approaches to inform host cellular effects on Wolbachia abundance in Drosophila
Source: Front Microbiol. 2024 Mar 25;15:1364009. doi: 10.3389/fmicb.2024.1364009 (PMC10999648; doi:10.3389/fmicb.2024.1364009)
Supplement: Supplementary file 1 [file Data_Sheet_1.zip › Additional File 1. References cited in Table S4.DOCX]

**Additional File 1. References cited in Table S4**

Agaisse, H., Burrack, L. S., Philips, J. A., Rubin, E. J., Perrimon, N., & Higgins, D. E. (2005). Genome-wide RNAi screen for host factors required for intracellular bacterial infection. *Science (New York, N.Y.)*, *309*(5738), 1248–1251. https://doi.org/10.1126/science.1116008

Alhazmi, A. (2018). Spleen Tyrosine Kinase as a Target Therapy for Pseudomonas aeruginosa Infection. *Journal of Innate Immunity*, *10*(4), 255–263. https://doi.org/10.1159/000489863

Ashida, H., Mimuro, H., Ogawa, M., Kobayashi, T., Sanada, T., Kim, M., & Sasakawa, C. (2011). Cell death and infection: A double-edged sword for host and pathogen survival. *The Journal of Cell Biology*, *195*(6), 931–942. https://doi.org/10.1083/jcb.201108081

Banerjee, R., Anguita, J., & Fikrig, E. (2000). Granulocytic Ehrlichiosis in Mice Deficient in Phagocyte Oxidase or Inducible Nitric Oxide Synthase. *Infection and Immunity*, *68*(7), 4361–4362.

Bechelli, J., Vergara, L., Smalley, C., Buzhdygan, T. P., Bender, S., Zhang, W., Liu, Y., Popov, V. L., Wang, J., Garg, N., Hwang, S., Walker, D. H., & Fang, R. (2019). Atg5 Supports Rickettsia australis Infection in Macrophages In Vitro and In Vivo. *Infection and Immunity*, *87*(1). https://doi.org/10.1128/IAI.00651-18

Birmingham, C. L., Smith, A. C., Bakowski, M. A., Yoshimori, T., & Brumell, J. H. (2006). Autophagy controls Salmonella infection in response to damage to the Salmonella-containing vacuole. *The Journal of Biological Chemistry*, *281*(16), 11374–11383. https://doi.org/10.1074/jbc.M509157200

Carroll, E. E. M., Wang, X., Shaw, D. K., O’Neal, A. J., Chávez, A. S. O., Brown, L. J., Boradia, V. M., Hammond, H. L., & Pedra, J. H. F. (2019). P47 licenses activation of the immune deficiency pathway in the tick Ixodes scapularis. *Proceedings of the National Academy of Sciences*, *116*(1), 205–210. https://doi.org/10.1073/pnas.1808905116

Cole, C., Thomas, S., Filak, H., Henson, P. M., & Lenz, L. L. (2012). Nitric Oxide Increases Susceptibility of Toll-like Receptor-Activated Macrophages to Spreading Listeria monocytogenes. *Immunity*, *36*(5), 807–820. https://doi.org/10.1016/j.immuni.2012.03.011

Colonne, P. M., Eremeeva, M. E., & Sahni, S. K. (2011). Beta Interferon-Mediated Activation of Signal Transducer and Activator of Transcription Protein 1 Interferes with Rickettsia conorii Replication in Human Endothelial Cells. *Infection and Immunity*, *79*(9), 3733–3743. https://doi.org/10.1128/IAI.05008-11

Czyż, D. M., Jain-Gupta, N., Shuman, H. A., & Crosson, S. (2016). A dual-targeting approach to inhibit *Brucella abortus* replication in human cells. *Scientific Reports*, *6*, 35835. https://doi.org/10.1038/srep35835

Czyż, D. M., Potluri, L.-P., Jain-Gupta, N., Riley, S. P., Martinez, J. J., Steck, T. L., Crosson, S., Shuman, H. A., & Gabay, J. E. (2014). Host-Directed Antimicrobial Drugs with Broad-Spectrum Efficacy against Intracellular Bacterial Pathogens. *MBio*, *5*(4). https://doi.org/10.1128/mBio.01534-14

Derré, I., Pypaert, M., Dautry-Varsat, A., & Agaisse, H. (2007). RNAi Screen in Drosophila Cells Reveals the Involvement of the Tom Complex in Chlamydia Infection. *PLoS Pathogens*, *3*(10). https://doi.org/10.1371/journal.ppat.0030155

Ferree, P. M., Frydman, H. M., Li, J. M., Cao, J., Wieschaus, E., & Sullivan, W. (2005). Wolbachia Utilizes Host Microtubules and Dynein for Anterior Localization in the Drosophila Oocyte. *PLoS Pathogens*, *1*(2). https://doi.org/10.1371/journal.ppat.0010014

Foldenauer, M. E. B., McClellan, S. A., Berger, E. A., & Hazlett, L. D. (2013). Mammalian Target of Rapamycin Regulates IL-10 and Resistance to Pseudomonas aeruginosa Corneal Infection. *The Journal of Immunology*, *190*(11), 5649–5658. https://doi.org/10.4049/jimmunol.1203094

Grobler, Y., Yun, C. Y., Kahler, D. J., Bergman, C. M., Lee, H., Oliver, B., & Lehmann, R. (2018). Whole genome screen reveals a novel relationship between Wolbachia levels and Drosophila host translation. *PLoS Pathogens*, *14*(11), e1007445. https://doi.org/10.1371/journal.ppat.1007445

Gross, A., Terraza, A., Ouahrani-Bettache, S., Liautard, J.-P., & Dornand, J. (2000). In Vitro Brucella suis Infection Prevents the Programmed Cell Death of Human Monocytic Cells. *Infection and Immunity*, *68*(1), 342–351.

Gutierrez, M. G., Master, S. S., Singh, S. B., Taylor, G. A., Colombo, M. I., & Deretic, V. (2004). Autophagy is a defense mechanism inhibiting BCG and Mycobacterium tuberculosis survival in infected macrophages. *Cell*, *119*(6), 753–766. https://doi.org/10.1016/j.cell.2004.11.038

Herren, J. K., & Lemaitre, B. (2011). Spiroplasma and host immunity: Activation of humoral immune responses increases endosymbiont load and susceptibility to certain Gram-negative bacterial pathogens in Drosophila melanogaster. *Cellular Microbiology*, *13*(9), 1385–1396. https://doi.org/10.1111/j.1462-5822.2011.01627.x

Jiménez de Bagüés, M. P., Gross, A., Terraza, A., & Dornand, J. (2005). Regulation of the Mitogen-Activated Protein Kinases by Brucella spp. Expressing a Smooth and Rough Phenotype: Relationship to Pathogen Invasiveness. *Infection and Immunity*, *73*(5), 3178–3183. https://doi.org/10.1128/IAI.73.5.3178-3183.2005

Kessler, M., Zielecki, J., Thieck, O., Mollenkopf, H.-J., Fotopoulou, C., & Meyer, T. F. (2012). Chlamydia trachomatis disturbs epithelial tissue homeostasis in fallopian tubes via paracrine Wnt signaling. *The American Journal of Pathology*, *180*(1), 186–198. https://doi.org/10.1016/j.ajpath.2011.09.015

Kim, D. H., Lim, J. J., Lee, J. J., Kim, D. G., Lee, H. J., Min, W., Kim, K. D., Chang, H. H., Rhee, M. H., Watarai, M., & Kim, S. (2012). Identification of genes contributing to the intracellular replication of Brucella abortus within HeLa and RAW 264.7 cells. *Veterinary Microbiology*, *158*(3–4), 322–328. https://doi.org/10.1016/j.vetmic.2012.02.019

Kintner, J., Moore, C. G., Whittimore, J. D., Butler, M., & Hall, J. V. (2017). Inhibition of Wnt Signaling Pathways Impairs Chlamydia trachomatis Infection in Endometrial Epithelial Cells. *Frontiers in Cellular and Infection Microbiology*, *7*, 501. https://doi.org/10.3389/fcimb.2017.00501

Kühbacher, A., Emmenlauer, M., Rämo, P., Kafai, N., Dehio, C., Cossart, P., & Pizarro-Cerdá, J. (2015). Genome-Wide siRNA Screen Identifies Complementary Signaling Pathways Involved in Listeria Infection and Reveals Different Actin Nucleation Mechanisms during Listeria Cell Invasion and Actin Comet Tail Formation. *MBio*, *6*(3), e00598-00515. https://doi.org/10.1128/mBio.00598-15

Kuijl, C., Savage, N. D. L., Marsman, M., Tuin, A. W., Janssen, L., Egan, D. A., Ketema, M., van den Nieuwendijk, R., van den Eeden, S. J. F., Geluk, A., Poot, A., van der Marel, G., Beijersbergen, R. L., Overkleeft, H., Ottenhoff, T. H. M., & Neefjes, J. (2007). Intracellular bacterial growth is controlled by a kinase network around PKB/AKT1. *Nature*, *450*(7170), 725–730. https://doi.org/10.1038/nature06345

Lad, S. P., Fukuda, E. Y., Li, J., Maza, L. M. de la, & Li, E. (2005). Up-Regulation of the JAK/STAT1 Signal Pathway during Chlamydia trachomatis Infection. *The Journal of Immunology*, *174*(11), 7186–7193. https://doi.org/10.4049/jimmunol.174.11.7186

Levenhagen, M. A., Alves, R. N., Rieck, S. E., Labruna, M. B., & Beletti, M. E. (2012). The role of cytoskeleton, components of inositol phospholipid signaling pathway and iron in <Emphasis Type="Italic">Ehrlichia canis</Emphasis> in vitro proliferation. *Veterinary Research Communications*, *36*(3), 195–199. https://doi.org/10.1007/s11259-012-9525-y

Limmer, S., Haller, S., Drenkard, E., Lee, J., Yu, S., Kocks, C., Ausubel, F. M., & Ferrandon, D. (2011). Pseudomonas aeruginosa RhlR is required to neutralize the cellular immune response in a Drosophila melanogaster oral infection model. *Proceedings of the National Academy of Sciences*, *108*(42), 17378–17383. https://doi.org/10.1073/pnas.1114907108

Lin, M., Zhu, M. X., & Rikihisa, Y. (2002). Rapid Activation of Protein Tyrosine Kinase and Phospholipase C-γ2 and Increase in Cytosolic Free Calcium Are Required by Ehrlichia chaffeensis for Internalization and Growth in THP-1 Cells. *Infection and Immunity*, *70*(2), 889–898. https://doi.org/10.1128/IAI.70.2.889-898.2002

Lina, T. T., Luo, T., Velayutham, T.-S., Das, S., & McBride, J. W. (2017). Ehrlichia Activation of Wnt-PI3K-mTOR Signaling Inhibits Autolysosome Generation and Autophagic Destruction by the Mononuclear Phagocyte. *Infection and Immunity*, *85*(12). https://doi.org/10.1128/IAI.00690-17

Liu, L., Dai, J., Zhao, Y. O., Narasimhan, S., Yang, Y., Zhang, L., & Fikrig, E. (2012). Ixodes scapularis JAK-STAT pathway regulates tick antimicrobial peptides, thereby controlling the agent of human granulocytic anaplasmosis. *The Journal of Infectious Diseases*, *206*(8), 1233–1241. https://doi.org/10.1093/infdis/jis484

Luo, T., Dunphy, P. S., Lina, T. T., & McBride, J. W. (2016). Ehrlichia chaffeensis Exploits Canonical and Noncanonical Host Wnt Signaling Pathways To Stimulate Phagocytosis and Promote Intracellular Survival. *Infection and Immunity*, *84*(3), 686–700. https://doi.org/10.1128/IAI.01289-15

Luo, T., Dunphy, P. S., & McBride, J. W. (2017). Ehrlichia chaffeensis Tandem Repeat Effector Targets Differentially Influence Infection. *Frontiers in Cellular and Infection Microbiology*, *7*. https://doi.org/10.3389/fcimb.2017.00178

Newton, I. L. G., & Sheehan, K. B. (2015). Passage of Wolbachia pipientis through Mutant Drosophila melanogaster Induces Phenotypic and Genomic Changes. *Applied and Environmental Microbiology*, *81*(3), 1032–1037. https://doi.org/10.1128/AEM.02987-14

Niu, H., Xiong, Q., Yamamoto, A., Hayashi-Nishino, M., & Rikihisa, Y. (2012). Autophagosomes induced by a bacterial Beclin 1 binding protein facilitate obligatory intracellular infection. *Proceedings of the National Academy of Sciences*, *109*(51), 20800–20807. https://doi.org/10.1073/pnas.1218674109

Niu, H., Yamaguchi, M., & Rikihisa, Y. (2008). Subversion of cellular autophagy by Anaplasma phagocytophilum. *Cellular Microbiology*, *10*(3), 593–605. https://doi.org/10.1111/j.1462-5822.2007.01068.x

Philips, J. A., Rubin, E. J., & Perrimon, N. (2005). Drosophila RNAi screen reveals CD36 family member required for mycobacterial infection. *Science (New York, N.Y.)*, *309*(5738), 1251–1253. https://doi.org/10.1126/science.1116006

Rennoll, S. A., Rennoll-Bankert, K. E., Guillotte, M. L., Lehman, S. S., Driscoll, T. P., Beier-Sexton, M., Rahman, M. S., Gillespie, J. J., & Azad, A. F. (2017). The Cat Flea (Ctenocephalides felis) Immune Deficiency Signaling Pathway Regulates Rickettsia typhi Infection. *Infection and Immunity*, *86*(1), e00562-17. https://doi.org/10.1128/IAI.00562-17

Rikihisa, Y., Zhang, Y., & Park, J. (1994). Inhibition of infection of macrophages with Ehrlichia risticii by cytochalasins, monodansylcadaverine, and taxol. *Infection and Immunity*, *62*(11), 5126–5132.

Rikihisa, Y., Zhang, Y., & Park, J. (1995). Role of Ca2+ and calmodulin in ehrlichial infection in macrophages. *Infection and Immunity*, *63*(6), 2310–2316.

Rogan, M. R., Patterson, L. L., Wang, J. Y., & McBride, J. W. (2019). Bacterial Manipulation of Wnt Signaling: A Host-Pathogen Tug-of-Wnt. *Frontiers in Immunology*, *10*. https://doi.org/10.3389/fimmu.2019.02390

Serbus, L. R., Ferreccio, A., Zhukova, M., McMorris, C. L., Kiseleva, E., & Sullivan, W. (2011). A feedback loop between Wolbachia and the Drosophila gurken mRNP complex influences Wolbachia titer. *Journal of Cell Science*, *124*(Pt 24), 4299–4308. https://doi.org/10.1242/jcs.092510

Severo, M. S., Choy, A., Stephens, K. D., Sakhon, O. S., Chen, G., Chung, D.-W. D., Le Roch, K. G., Blaha, G., & Pedra, J. H. F. (2013). The E3 ubiquitin ligase XIAP restricts Anaplasma phagocytophilum colonization of Ixodes scapularis ticks. *The Journal of Infectious Diseases*, *208*(11), 1830–1840. https://doi.org/10.1093/infdis/jit380

Steele, S., Brunton, J., Ziehr, B., Taft-Benz, S., Moorman, N., & Kawula, T. (2013). Francisella tularensis Harvests Nutrients Derived via ATG5-Independent Autophagy to Support Intracellular Growth. *PLOS Pathogens*, *9*(8), e1003562. https://doi.org/10.1371/journal.ppat.1003562

Turco, J., Liu, H., Gottlieb, S. F., & Winkler, H. H. (1998). Nitric oxide-mediated inhibition of the ability of Rickettsia prowazekii to infect mouse fibroblasts and mouse macrophagelike cells. *Infection and Immunity*, *66*(2), 558–566.

Walker, D. H., Firth, W. T., Ballard, J. G., & Hegarty, B. C. (1983). Role of Phospholipase-Associated Penetration Mechanism in Cell Injury by Rickettsia rickettsii. *Infection and Immunity*, *40*(2), 840–842.

Wang, J., Wu, Y., Yang, G., & Aksoy, S. (2009). Interactions between mutualist Wigglesworthia and tsetse peptidoglycan recognition protein (PGRP-LB) influence trypanosome transmission. *Proceedings of the National Academy of Sciences*, *106*(29), 12133–12138. https://doi.org/10.1073/pnas.0901226106

Wang, J., Yang, K., Zhou, L., MinhaoWu, Wu, Y., Zhu, M., Lai, X., Chen, T., Feng, L., Li, M., Huang, C., Zhong, Q., & Huang, X. (2013). MicroRNA-155 Promotes Autophagy to Eliminate Intracellular Mycobacteria by Targeting Rheb. *PLOS Pathogens*, *9*(10), e1003697. https://doi.org/10.1371/journal.ppat.1003697

Wei, P., Cui, G., Lu, Q., Yang, L., Guan, Z., Sun, W., Zhao, Y., Wang, S., & Peng, Q. (2015). A20 promotes Brucella intracellular growth via inhibition of macrophage cell death and activation. *Veterinary Microbiology*, *175*(1), 50–57. https://doi.org/10.1016/j.vetmic.2014.11.006

White, P. M., Serbus, L. R., Debec, A., Codina, A., Bray, W., Guichet, A., Lokey, R. S., & Sullivan, W. (2017). Reliance of Wolbachia on High Rates of Host Proteolysis Revealed by a Genome-Wide RNAi Screen of Drosophila Cells. *Genetics*, *205*(4), 1473–1488. https://doi.org/10.1534/genetics.116.198903

Xiong, Q., Lin, M., & Rikihisa, Y. (2009). Cholesterol-Dependent Anaplasma phagocytophilum Exploits the Low-Density Lipoprotein Uptake Pathway. *PLoS Pathogens*, *5*(3). https://doi.org/10.1371/journal.ppat.1000329

Yang, L., Liu, C., Zhao, W., He, C., Ding, J., Dai, R., Xu, K., Xiao, L., Luo, L., Liu, S., Li, W., & Meng, H. (2018). Impaired Autophagy in Intestinal Epithelial Cells Alters Gut Microbiota and Host Immune Responses. *Applied and Environmental Microbiology*, *84*(18). https://doi.org/10.1128/AEM.00880-18

Yuan, K., Huang, C., Fox, J., Laturnus, D., Carlson, E., Zhang, B., Yin, Q., Gao, H., & Wu, M. (2012). Autophagy plays an essential role in the clearance of Pseudomonas aeruginosa by alveolar macrophages. *Journal of Cell Science*, *125*(Pt 2), 507–515. https://doi.org/10.1242/jcs.094573

Zhang, Q., Wang, C., Liu, Z., Liu, X., Han, C., Cao, X., & Li, N. (2012). Notch signal suppresses Toll-like receptor-triggered inflammatory responses in macrophages by inhibiting extracellular signal-regulated kinase 1/2-mediated nuclear factor κB activation. *The Journal of Biological Chemistry*, *287*(9), 6208–6217. https://doi.org/10.1074/jbc.M111.310375

Zhang, Y., & Rikihisa, Y. (1997). Tyrosine phosphorylation is required for ehrlichial internalization and replication in P388D1 cells. *Infection and Immunity*, *65*(7), 2959–2964.

Zhu, B., Das, S., Mitra, S., Farris, T. R., & McBride, J. W. (2017). Ehrlichia chaffeensis TRP120 Moonlights as a HECT E3 Ligase Involved in Self- and Host Ubiquitination To Influence Protein Interactions and Stability for Intracellular Survival. *Infection and Immunity*, *85*(9). https://doi.org/10.1128/IAI.00290-17
