# Supplementary material for: Cross-validation of chemical and genetic disruption approaches to inform host cellular effects on Wolbachia abundance in Drosophila
Source: Front Microbiol. 2024 Mar 25;15:1364009. doi: 10.3389/fmicb.2024.1364009 (PMC10999648; doi:10.3389/fmicb.2024.1364009)
Supplement: Supplementary file 1 [file Data_Sheet_1.zip › Figure S1, S2.PPTX]

## Slide 1
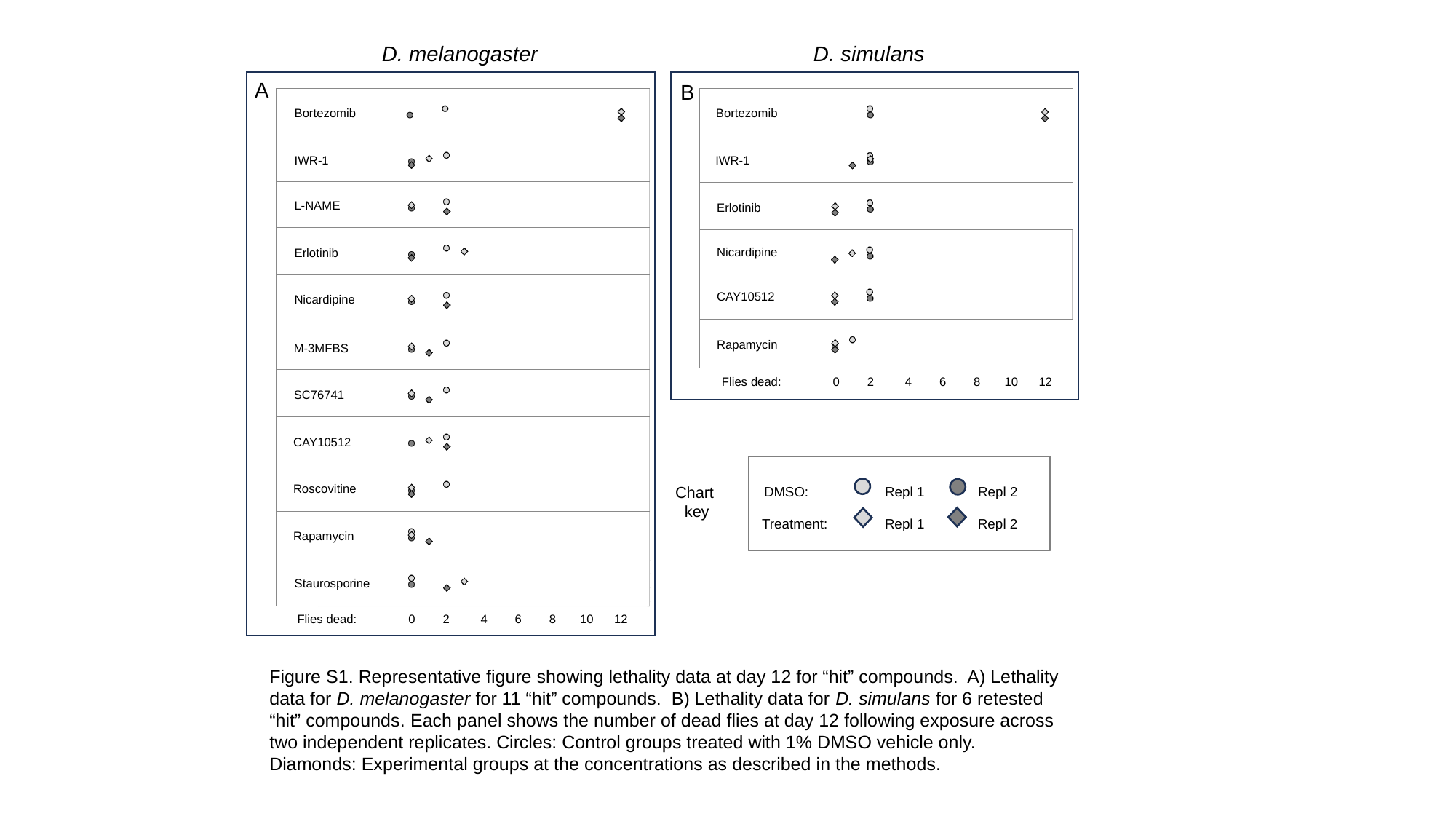

D. melanogaster                                              D. simulans
A
Bortezomib
IWR-1
L-NAME
Erlotinib
Nicardipine
M-3MFBS
SC76741
CAY10512
Roscovitine
Rapamycin
Staurosporine
Flies dead: 0 2 4 6 8 10 12
B
Bortezomib
IWR-1
Erlotinib
Nicardipine
CAY10512
Rapamycin
Flies dead: 0 2 4 6 8 10 12
Chart
key
Treatment: Repl 1 Repl 2
DMSO: Repl 1 Repl 2
Figure S1. Representative figure showing lethality data at day 12 for “hit” compounds.  A) Lethality data for D. melanogaster for 11 “hit” compounds.  B) Lethality data for D. simulans for 6 retested “hit” compounds. Each panel shows the number of dead flies at day 12 following exposure across two independent replicates. Circles: Control groups treated with 1% DMSO vehicle only. Diamonds: Experimental groups at the concentrations as described in the methods.

## Slide 2
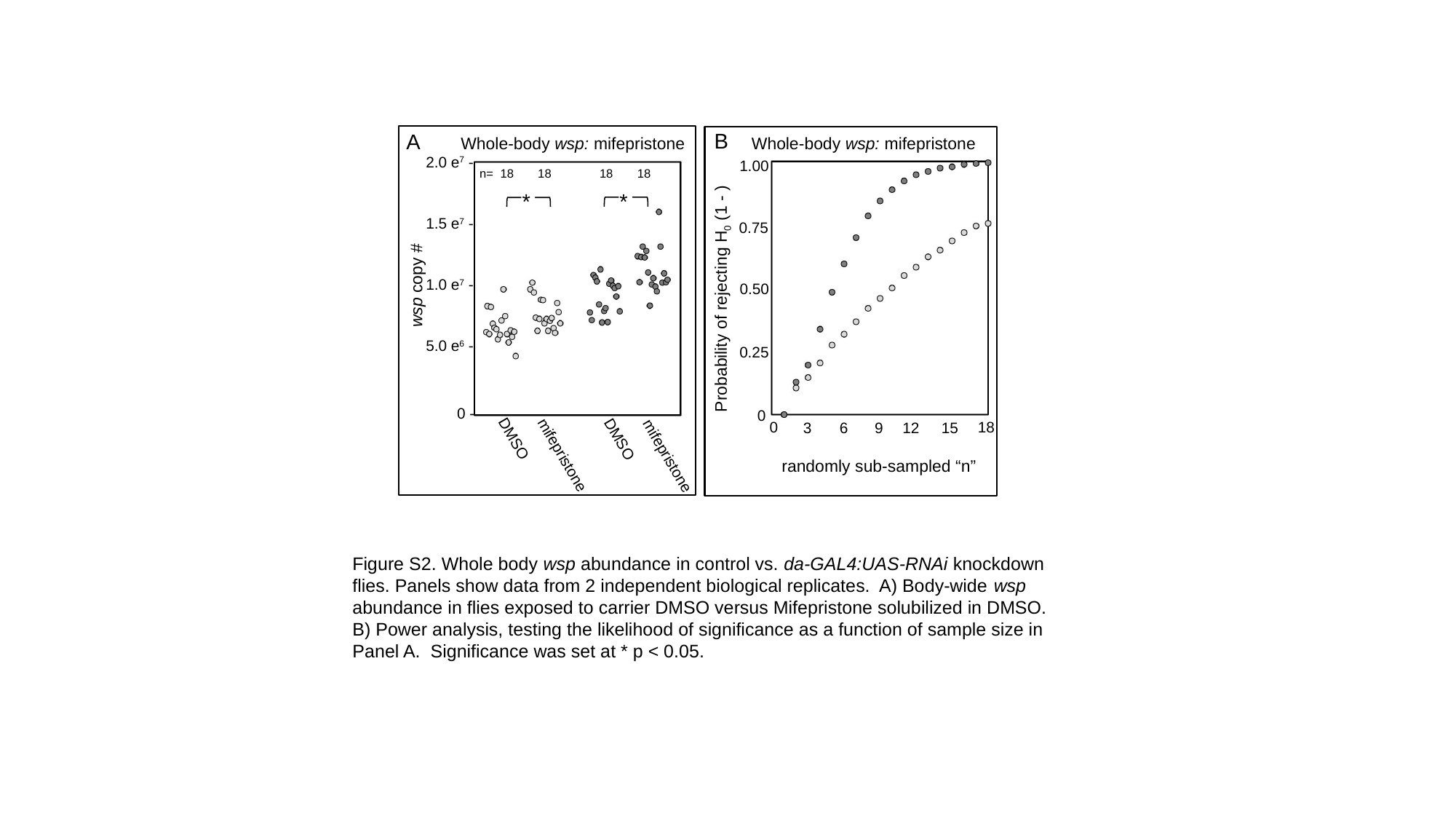

B
Whole-body wsp: mifepristone
1.00
0.75
0.50
0.25
0
0
18
12
15
3
6
9
randomly sub-sampled “n”
A
Whole-body wsp: mifepristone
n= 18 18 18 18
*
*
wsp copy #
0 -
DMSO
DMSO
mifepristone
mifepristone
Figure S2. Whole body wsp abundance in control vs. da-GAL4:UAS-RNAi knockdown flies. Panels show data from 2 independent biological replicates.  A) Body-wide wsp abundance in flies exposed to carrier DMSO versus Mifepristone solubilized in DMSO.  B) Power analysis, testing the likelihood of significance as a function of sample size in Panel A.  Significance was set at * p < 0.05.
2.0 e7 -
1.5 e7 -
1.0 e7 -
5.0 e6 -
